# Assessment of Surface Roughness and Bacterial Adhesion of Occlusal Splints Fabricated with Different Layer Thicknesses, Polishing Techniques and Build Orientations

**DOI:** 10.3390/polym18121545

**Published:** 2026-06-22

**Authors:** Merve Dede, Sina Saygili, Nursen Topcuoglu

**Affiliations:** 1 Department of Prosthodontics, Faculty of Dentistry, Istanbul Galata University, Istanbul 34430, Turkey; 2 Department of Prosthodontics, Faculty of Dentistry, Istanbul University, Istanbul 34093, Turkey; sinasaygili@istanbul.edu.tr; 3 Department of Basic Medical Sciences, Faculty of Dentistry, Istanbul University, Istanbul 34098, Turkey; nurtopcu@istanbul.edu.tr

**Keywords:** DLP printing, occlusal splint resin, surface roughness, *Streptococcus mutans*, additive manufacturing, bacterial adhesion

## Abstract

This study evaluated the combined effects of build orientation, layer thickness, and polishing protocols on surface roughness and bacterial adhesion of occlusal splints. Ten disc-shaped specimens (Ø16 × 3 mm) were fabricated for each group using a digital light processing (DLP)-based 3D printer. Specimens were printed at two orientations (0° and 90°) and two layer thicknesses (50 and 100 µm) using a splint resin. Surface roughness was measured with a contact profilometer, and bacterial adhesion was measured by optical density (OD) readouts for *Streptococcus mutans* using a spectrophotometer. Surface morphology was examined by field-emission scanning electron microscopy (SEM). Statistical analyses were performed using jamovi. Because normality and/or homogeneity assumptions were not met, robust analysis of variance was applied. Polishing protocol significantly affected surface roughness (Ra) values. Unpolished specimens showed the highest Ra values, whereas mechanical polishing combined with centrifugation produced the lowest values. No significant main effects of polishing protocol, layer thickness or orientation were observed for bacterial adhesion. SEM findings supported the roughness results. Surface roughness was primarily influenced by polishing protocols and their interactions, whereas bacterial adhesion remained relatively stable. The weak Ra–OD correlation indicated that surface roughness alone was not a reliable predictor of bacterial adhesion.

## 1. Introduction

Temporomandibular disorders and associated joint pain are frequently managed with occlusal splints [1]. Intraoral occlusal splints are fabricated to provide uniform and balanced occlusal contacts without producing permanent alterations in the mandibular rest position or dental occlusion. A properly designed splint contributes to a harmonious functional relationship among the masticatory muscles, disc assemblies, joints, ligaments, bone, teeth, and associated supporting structures [2].

Occlusal splints are typically constructed to cover the occlusal and incisal surfaces of the maxillary and/or mandibular teeth [3]. It has been demonstrated that manufacturing methods can influence the mechanical properties of dental materials [4].

Accordingly, it can be assumed that fabrication techniques could also affect bacterial adhesion. Oral biofilm formation is a sequential process that begins with the adhesion of early colonizers, such as streptococci, to the pellicle-coated surface and is markedly influenced by material-dependent surface characteristics [5]. Subsequently, bacterial accumulation and biofilm maturation can contribute to the onset of dental caries and periodontal inflammation [6,7]. Surface characteristics such as roughness play a critical role in bacterial adhesion. The general consensus is that low surface roughness may help minimize biofilm accumulation [8].

Polymethyl methacrylate (PMMA) is the most commonly used material for the fabrication of occlusal splints [9]. These devices can be fabricated using conventional techniques [10,11] as well as computer-aided design/computer-aided manufacturing (CAD/CAM) systems, following the emergence of digital technologies. Until recently, occlusal splints were manufactured exclusively through subtractive techniques; however, additive manufacturing (AM) technologies have now become available as an alternative [9]. Patient-specific devices can be fabricated with three-dimensional (3D) printing by a layer-by-layer approach [12,13]. AM technologies are increasingly favoured because of several advantages, including faster production, reduced material waste, cost-effectiveness, and high accuracy [13,14].

Although additive manufacturing offers an efficient approach for producing occlusal splints, the layer-by-layer deposition process may adversely affect surface integrity by generating staircase-like surface features that contribute to increased roughness [15,16,17,18]. Surface roughness is a critical characteristic because values above 0.2 μm have been linked to enhanced microbial colonization [19], while surface irregularities of approximately 0.5 μm may be detectable by patients during intraoral use [20].

The manufacturing accuracy and mechanical properties of AM dental devices have been reported to be influenced by the type of AM technology, printer, and material used [21,22], as well as by printing parameters such as layer thickness [23,24,25], print orientation [26,27,28,29] and position on the build platform [30], polishing [31], and post-processing methods [30,32]. Post-processing represents a fundamental stage in the fabrication of 3D-printed devices and plays a significant role in determining their final properties. Common post-processing procedures include cleaning, post-curing, and various surface finishing methods, such as polishing, resin application, and glazing [21,33]. Different surface finishing techniques, including polishing, resin coating, and glaze coating, have been suggested as effective strategies for improving the surface characteristics of additively manufactured materials [34,35,36]. Previous investigations have assessed the influence of polishing and resin-coating protocols on 3D-printed occlusal splint materials [15,16,33,37]. Nevertheless, limited information is available regarding the combined effects of surface finishing methods and post-curing conditions on the surface properties of 3D-printed splint resins.

We proposed a null hypothesis wherein build orientation, layer thickness, and polishing protocols would not exert a statistically significant influence on the surface characteristics of 3D-printed occlusal splint resins or on *Streptococcus mutans* adhesion, and there would be no correlation between Ra and OD.

## 2. Materials and Methods

### 2.1. Oral Splint Materials and Manufacturing of the Discs

In the present study, ten disc-shaped specimens (Ø16 × 3 mm) for each group—a total of 160 disc-shaped specimens—were manufactured using 3D-modelling software (Meshmixer 3.5.474, Autodesk, San Rafael, CA, USA) and exported in standard tessellation language (STL) format. The STL files were imported into the printer’s slicing software, and the specimens were manufactured using a digital light processing (DLP)-based 3D printer (Max UV, Asiga, Australia). Half of the specimens were positioned at a 90° orientation, and the other half of the specimens were positioned at a 0° orientation relative to the build platform, and support structures were generated automatically. Printing was carried out using two different layer thicknesses (50 µm and 100 µm). A splint resin (Dentafab Powerresins, Istanbul, Turkey) was selected for additive manufacturing.

Following fabrication, post-processing procedures were carried out in accordance with the manufacturer’s recommendations. In the Alcohol groups, the 3D-printed splint specimens initially underwent cleaning in an ultrasonic bath (Sonorex Super RK100H, Bandelin, Germany) containing 99% isopropanol for 3 min. In the centrifuge group, excess resin was removed by centrifugation at 500 rpm for 3 min, using an in-house centrifuge device.

Each group was divided into four subgroups (n = 10 per group) according to the polishing method: A + M (Alcohol + Mechanical Polishing), A + RC (Alcohol + Resin Coating), A + NP (Alcohol + No Polishing), C + M (Centrifuge + Mechanical Polishing) (Table 1).

In the Alcohol + Mechanical Polishing group, excess resin was removed with alcohol prior to mechanical polishing. Mechanical polishing was then carried out using felt wheels and cotton brushes.

In the Alcohol + Resin Coating group, excess resin was removed with alcohol prior to resin coating. Then, a resin coating was applied to the specimen surface. The coated specimens underwent post-curing in ambient air at 60 °C for 60 min using the curing unit.

In the Alcohol + No Polishing group, excess resin was only removed with alcohol.

In the Centrifuge + Mechanical Polishing group, excess resin was removed by centrifugation at 500 rpm for 3 min using an in-house centrifuge device. Then, mechanical polishing was carried out using felt wheels and cotton brushes.

Surface roughness measurements were performed on the fabricated disc-shaped specimens using a contact profilometer (Mitutoyo SJ-400, Mitutoyo Corp., Kawasaki, Japan). Three measurements were performed for each specimen, and the mean surface roughness (Ra) was calculated. The profilometer was calibrated using a reference block before measurements were obtained for each group. For this study, disc-shaped specimens were used for microbiological evaluations and scanning electron microscopy (SEM) imaging.

The groups were assigned codes according to their production methods: Group 1: 50H-A (50 µm, horizontal, Alcohol + Mechanical Polishing); Group 2: 100H-A (100 µm, horizontal, Alcohol + Mechanical Polishing); Group 3: 50H-B (50 µm, horizontal, Alcohol + Resin Coating); Group 4: 100H-B (100 µm, horizontal, Alcohol + Resin Coating); Group 5: 50H-C (50 µm, horizontal, Alcohol + No Polishing); Group 6: 100H-C (50 µm, horizontal Alcohol + No Polishing); Group 7: 50H-D: (50 µm, horizontal, Centrifuge + Mechanical Polishing); Group 8: 100H-D (100 µm, horizontal, Centrifuge + Mechanical Polishing); Group 9: 50V-A (50 µm, vertical, Alcohol + Mechanical Polishing); Group 10: 100V-A (100 µm, vertical, Alcohol + Mechanical Polishing); Group 11: 50V-B (50 µm, vertical, Alcohol + Resin Coating); Group 12: 100V-B (100 µm, vertical, Alcohol + Resin Coating); Group 13: 50V-C (50 µm, vertical, Alcohol + No Polishing); Group 14: 100V-C (50 µm, vertical, Alcohol + No Polishing); Group 15: 50V-D: (50 µm, vertical, Centrifuge + Mechanical Polishing); Group 16: 100V-D (100 µm, vertical, Centrifuge + Mechanical Polishing).

### 2.2. Assessment of Bacterial Adhesion

Disc specimens were individually packaged and sterilized using hydrogen gas plasma (STERRAD^®^ System, Advanced Sterilization Products, Irvine, CA, USA) at 50 °C for 50 min prior to bacterial testing (n = 10 per group).

#### 2.2.1. Bacterial Strain and Culture Conditions

*Streptococcus mutans* ATCC 25175 was selected as the test organism. Cultures were maintained by overnight subculture on Brain Heart Infusion Agar (BHI, Merck KGaA 64271 Darmstadt, Germany) at 37 °C under anaerobic conditions (80% N_2_, 10% CO_2_, and 10% H_2_) provided by an atmosphere generator (AnaeroGen, Oxoid, Basingstoke, UK) and incubation jar. A pre-culture was prepared by inoculating a single bacterial colony into 10 mL of BHI broth (Merck KGaA, Darmstadt, Germany), followed by overnight incubation at 37 °C under anaerobic conditions. The bacterial suspension was then obtained by diluting the pre-culture at a ratio of 1:10 in fresh BHI broth and incubating for an additional 1 h. The turbidity of the suspension was adjusted to the 0.5 McFarland standard (5 × 10^8^ CFU/mL), and diluted to 1:100 (approximately 10^6^ CFU/mL).

#### 2.2.2. Bacterial Adhesion Assay

The experiment was conducted using sterile 24-well culture plates (Isolab Laborgerate GmbH, Eschau, Germany), with each specimen aseptically placed at the bottom of an individual well. For pellicle formation, the fragments were coated with 500 µL of artificial saliva solution (Testonic, Colin Specific Solutions, Istanbul, Turkey) and incubated at 37 °C on a shaker for 1 h.

Following pellicle formation, the specimens were rinsed with 2 mL of phosphate-buffered saline (PBS) and transferred to new sterile plates. Each disc was then immersed in 1.6 mL of BHI broth supplemented with 5% sucrose and inoculated with 200 μL of the bacterial suspension. The plates were incubated anaerobically at 37 °C for 4 h.

After incubation, the discs were gently rinsed three times with physiological saline to remove non-adherent bacteria. Each specimen was then transferred into a sterile tube containing 1 mL of physiological saline and vortexed for 1 min to detach adherent bacteria. Subsequently, 20 μL of the resulting suspension was transferred into sterile 96-well microtiter plates containing 200 μL of BHI broth. Following anaerobic incubation at 37 °C for 24 h, the absorbance of the bacterial turbidity was monitored at 630 nm wavelength using a spectrophotometer (Maxilab Biotechnology MS4-MaxiRead96, Istanbul, Turkey). Data were recorded in optical density (OD) units. Automixing prior to each reading ensured a homogeneous bacterial cell suspension. The values of the negative control wells were considered as the baseline and were then subtracted from the respective experimental sets.

### 2.3. Scanning Electron Microscopy (SEM) Analysis

Specimens were mounted on aluminum stubs and coated with a 10–20 nm gold–palladium (Au–Pd) layer using a sputter coater (Leica EM ACE200, Leica Microsystems, Vienna, Austria) to enhance conductivity. Surface morphology was examined using a field-emission scanning electron microscope (Zeiss EVO 40, Carl Zeiss, Oberkochen, Germany) at an accelerating voltage of 10 kV and a working distance of 7–10 mm. Images were obtained using a secondary electron detector at ×1000 magnification.

### 2.4. Statistical Analysis

All statistical analyses were conducted using Jamovi software (Version 2.7.6.0). As the data did not fully meet normality and/or homogeneity of variance assumptions, robust analysis of variance (robust ANOVA) was employed.

The effects of build orientation, layer thickness, and cleaning protocol on surface roughness (Ra) and optical density (OD) were evaluated using robust ANOVA. The relationship between Ra and OD values across different regions was assessed using Spearman’s rank correlation coefficient. Statistical significance was set at α = 0.05 for all analyses.

## 3. Results

### 3.1. Surface Roughness

The effects of polishing method, layer thickness, and build orientation on surface roughness (Ra) were evaluated using robust ANOVA. The main effect of the polishing method was statistically significant (*p* < 0.001). Across all groups, the A + NP method resulted in the highest mean Ra values, whereas the C + M method consistently produced the lowest Ra values. Mechanical polishing and A + RC showed intermediate roughness levels.

The main effect of layer thickness was not statistically significant (*p* = 0.272), indicating that Ra values were comparable between the 50 µm and 100 µm layers, when averaged across polishing methods and orientations. Similarly, the main effect of orientation did not reach statistical significance (*p* = 0.063).

However, a statistically significant polishing × layer thickness interaction was observed (*p* < 0.001). This interaction revealed that the influence of the polishing method on Ra differed depending on layer thickness. In particular, the increase in Ra associated with the A + NP method was more pronounced at 100 µm compared with 50 µm, whereas C + M polishing maintained relatively low Ra values at both thicknesses.

**Figure 1:**
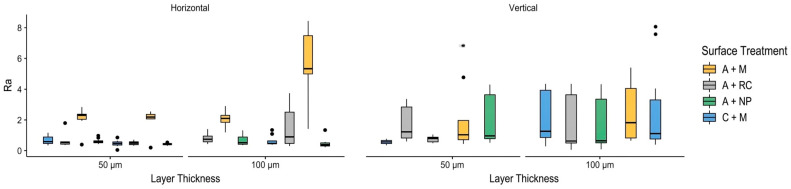
Surface roughness (Ra) values of samples produced by different additive manufacturing (AM) methods.

In addition, a significant polishing × layer thickness × orientation interaction was detected (*p* < 0.001). This finding indicates that surface roughness is not determined by a single factor alone, but rather by the combined effects of polishing protocol, layer thickness, and build orientation. Groups sharing the same interaction lettering were statistically similar, while those with different letters showed significant differences (Table 2) (Figure 1).

### 3.2. Bacterial Adhesion

**Figure 2:**
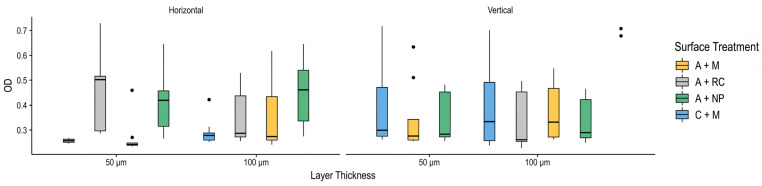
The distribution of mean OD630 readings for *S. mutans* in the subgroups for all occlusal splint specimens.

The mean optical density (OD) readings and the standard deviations determined for *S. mutans* adhesion in the subgroups of each material group and the comparison between the subgroups are presented in Table 3 and Figure 2. The main effects of polishing method (*p* = 0.078), layer thickness (*p* = 0.496), and orientation (*p* = 0.607) on OD values were not statistically significant. Mean OD values remained relatively stable across polishing protocols and thickness levels when these factors were considered independently.

Nevertheless, a significant polishing × orientation interaction was observed (*p* < 0.001), indicating that the effect of polishing on OD depended on the build orientation. Specifically, certain polishing methods yielded higher OD values in vertically oriented specimens compared with horizontally oriented ones.

The polishing × layer thickness interaction was not statistically significant (*p* = 0.105), and the layer thickness × orientation interaction was also not significant (*p* = 0.831). In contrast, the three-way interaction (polishing × layer thickness × orientation) was statistically significant (*p* < 0.001), demonstrating that subtle but meaningful changes in OD occur when all three parameters are considered simultaneously.

### 3.3. Correlation Between Surface Roughness and Bacterial Adhesion

Spearman correlation analysis revealed a weak negative correlation between Ra and OD values (r = −0.155). This correlation was at the threshold of statistical significance (*p* = 0.050), suggesting that increased surface roughness tends to be associated with slightly lower optical density; however, this relationship is weak and should be interpreted with caution (Table 4).

### 3.4. Surface Morphology

**Figure 3:**
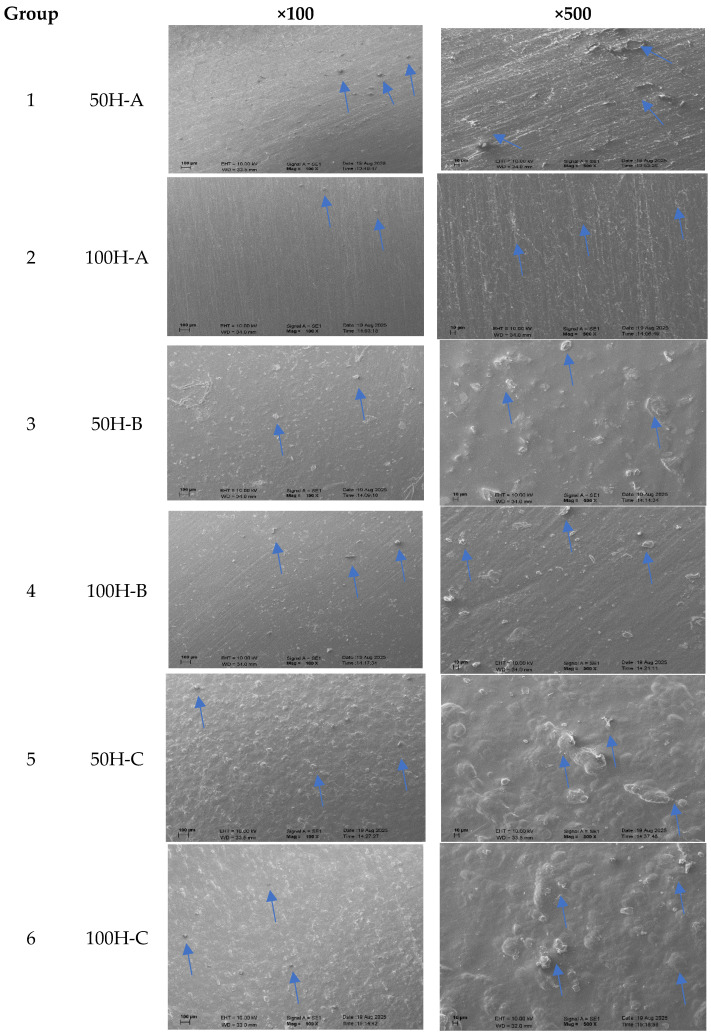

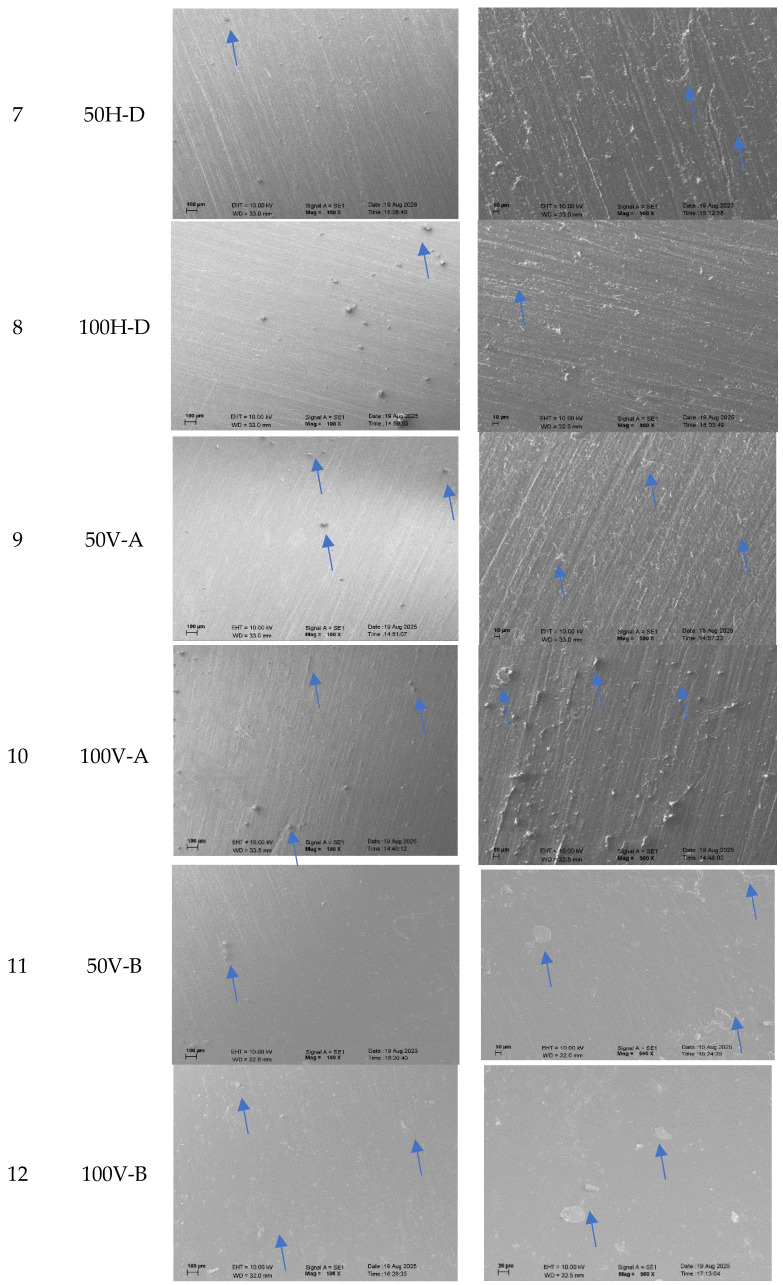

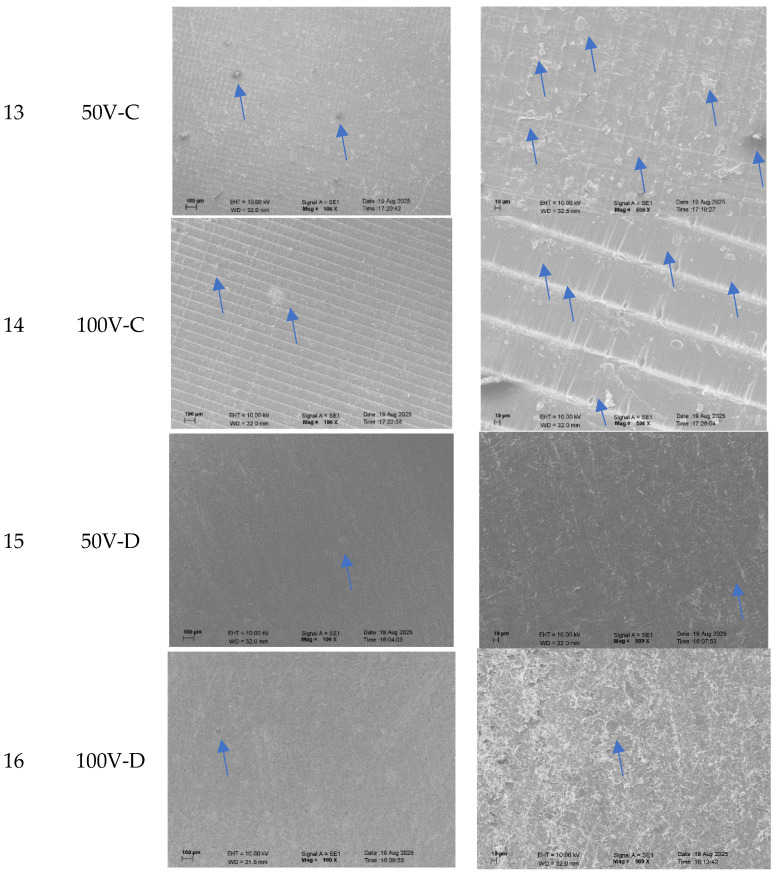
Representative SEM micrographs of the specimen surfaces are presented at ×100 and ×500 magnifications.

Representative SEM micrographs of the splint specimen surfaces are presented in Figure 3 at ×100 and ×500 magnification. The images revealed distinct differences in surface microstructure among the evaluated groups. The polished specimens exhibited the smoothest surfaces, with only minor scratches and surface irregularities observed. In contrast, the non-polished group displayed the most pronounced grooves and particulate features. Surface irregularities were noticeably more evident in the 100 µm groups compared with the 50 µm groups. Furthermore, significant surface irregularities were observed in the alcohol-cleaned groups.

Apart from minor surface imperfections, the vertical and horizontal printing groups of the printable materials exhibited similar surface morphologies in the SEM analysis (Figure 3).

## 4. Discussion

In this study, we aimed to evaluate the surface roughness of occlusal splint specimens fabricated using sixteen different additive manufacturing methods, as well as their bacterial adhesion. While the polishing method (*p* < 0.001) alone had a statistically significant effect on surface roughness, layer thickness (*p* = 0.272) and build orientation (*p* = 0.063) did not show a significant effect on surface roughness. The main effects of polishing method (*p* = 0.078), layer thickness (*p* = 0.496), and orientation (*p* = 0.607) on OD values were not statistically significant. Overall, these findings indicate that surface roughness is strongly influenced by polishing protocols and their interactions with layer thickness and orientation, whereas optical density is comparatively more stable, being affected mainly through interaction effects rather than main factors. The weak correlation between Ra and OD suggests that surface roughness alone is not a strong predictor of optical behaviour in this material system. Therefore, the null hypothesis of the study was partially rejected.

In recent years, additive dental materials have attracted growing attention in parallel with advances in digital manufacturing technologies. Previous studies have examined the effects of polymerization protocols, cleaning procedures, and various fabrication parameters on material properties such as surface roughness, surface free energy (SFE), and hardness [12,38,39,40,41]. Nevertheless, the influence of these variables on bacterial adhesion remains inadequately addressed, particularly with respect to *Candida albicans* and *Streptococcus mutans*. The use of a single or a limited number of microbial species has been the most frequently investigated, including *S. mutans* [42,43,44,45,46], *Streptococcus gordonii*, *Streptococcus oralis*, *Streptococcus sanguinis*, *Actinomyces naeslundii* [47], and *C. albicans* [48]. In the present study, *S. mutans* was specifically included to model a biofilm with cariogenic potential, given that dental caries represents one of the most common complications associated with the prolonged use of oral appliances.

In patients presenting with parafunctional activities such as bruxism, occlusal splints must exhibit adequate wear resistance and the ability to tolerate occlusal loading [49]. Surface roughness is a critical parameter that directly influences the wear resistance of materials and, consequently, their clinical performance [50]. Manufacturing parameters applied in additive manufacturing (AM) processes can affect the mechanical behaviour of materials, both directly and indirectly [21]. In the present study, the main effect of polishing method was statistically significant (*p* < 0.001). Across all groups, the A + NP method resulted in the highest mean Ra values, whereas the C + M method consistently produced the lowest Ra values. A + M and A + RC showed intermediate roughness levels. Polishing techniques reduce surface irregularities through controlled material removal, whereas coating procedures primarily act by filling in layer-induced defects, leading to surface smoothing without significant substrate loss [16,51]. Although surface coatings have been reported to enhance surface properties [16,34,36], their long-term durability remains questionable [52,53,54], as the applied layers may be prone to wear, delamination, and degradation under functional occlusal forces and routine cleaning protocols. These limitations may adversely affect the long-term clinical performance of coated surfaces. Furthermore, the majority of existing studies have predominantly investigated denture base resins [34,52,53], while evidence regarding the application and longevity of coating procedures in 3D-printed occlusal splints is still limited. However, cleaning durations may allow solvent molecules to penetrate into the material, potentially altering its physicochemical properties. Indeed, the formation of surface cracks has been documented following cleaning [55].

The main effect of layer thickness was not statistically significant (*p* = 0.272). However, a statistically significant polishing × layer thickness interaction was observed (*p* < 0.001). This interaction revealed that the influence of the polishing method on Ra differed depending on layer thickness. In particular, the increase in Ra associated with the A + NP method was more pronounced at 100 µm compared with 50 µm. Previous studies have commonly evaluated specimens produced at 25, 50, and 100 μm and have consistently demonstrated that wear resistance improves as layer thickness decreases, suggesting a beneficial effect of thinner layers on wear behaviour [56,57]. In vat polymerization systems, decreasing layer thickness has been reported to enhance mechanical properties [58]. Supporting this, another study assessing five different layer thicknesses similarly reported no significant effect on the mechanical properties of the materials [25].

Beyond mechanical performance, layer thickness also plays a role in determining material isotropy, an important quality attribute. Reducing layer thickness may enhance interlayer bonding, leading to a more uniform and homogeneous structure [59]. Although isotropy is governed by multiple factors, optimizing layer thickness toward lower values may provide certain advantages. As suggested by Simeon et al. [59], future research should consider the use of 3D printing systems capable of producing layers thinner than 50 μm to further investigate their impact on interlayer adhesion and anisotropy.

The main effect of orientation was not statistically significant (*p* = 0.063). However, a significant polishing × layer thickness × orientation interaction was detected (*p* < 0.001). This finding indicates that surface roughness is not determined by a single factor alone, but rather by the combined effects of polishing protocol, layer thickness, and build orientation.

The SEM images revealed distinct differences in surface microstructure among the evaluated groups. The polished specimens exhibited the smoothest surfaces, with only minor scratches and surface irregularities observed. In contrast, the non-polished group displayed the most pronounced grooves and particulate features. Although the main effect of layer thickness was not statistically significant (*p* = 0.272), the surface roughness of a 50 µm thickness (0.973 ± 0.086) was found to be lower than that of a 100 µm thickness (1.209 ± 0.195), and surface irregularities were noticeably more evident in the 100 µm groups compared with the 50 µm groups. Furthermore, significant surface irregularities were observed in the alcohol-cleaned groups. Cleaning solutions may permit solvent molecules to diffuse into the material, which can potentially modify its physicochemical properties [55], which is also consistent with the observations in the SEM images. Apart from minor surface imperfections, the vertical and horizontal printing groups of the printable materials exhibited similar surface morphologies in the SEM analysis. Although not statistically significant, vertically oriented fabrication tended to increase surface roughness (Figure 3).

The main effects of polishing method (*p* = 0.078), layer thickness (*p* = 0.496), and orientation (*p* = 0.607) on bacterial adhesion were not statistically significant. Overall, these findings indicate that surface roughness is strongly influenced by polishing protocols and their interactions with layer thickness and orientation, whereas bacterial adhesion is comparatively more stable and is affected mainly through interaction effects rather than individual main factors. As reported by Poker et al. [60], surface roughness was not found to have a statistically significant influence on *S. mutans* adhesion.

The influence of layer thickness on OD values was not statistically significant (*p* = 0.496). Polishing × layer thickness interaction (*p* = 0.105) and the layer thickness × orientation interaction were also not significant (*p* = 0.831). In the present study, although similar surface roughness was observed at layer thicknesses of 50 μm and 100 μm, specimens fabricated with a 50 μm layer thickness demonstrated lower bacterial adhesion [61].

The influence of orientation on OD values, which represent bacterial adhesion, was not statistically significant (*p* = 0.607). Horizontally fabricated additively manufactured materials specimens exhibited reduced bacterial adhesion compared with vertically fabricated specimens. In line with our findings, existing evidence suggests that variations in printing orientation (0°, 45°, and 90°) do not have a significant impact on microbial adhesion [62]. Also, several studies, consistent with the present findings, have reported no significant relationship between surface roughness and either bacterial adhesion or total biofilm mass of *S. mutans*, even when baseline Ra values differ substantially [63].

The absence of a significant correlation between Ra and OD values suggests that bacterial adhesion may be influenced by factors other than surface roughness alone. Previous studies have demonstrated that bacterial attachment is a multifactorial process affected not only by surface topography but also by physicochemical properties such as surface free energy, hydrophobicity, and material composition [64,65]. As a result, materials exhibiting similar roughness values may present different bacterial adhesion patterns because of variations in their surface chemistry [64]. Furthermore, in the oral environment, microorganisms adhere primarily to the acquired salivary pellicle rather than directly to the underlying material surface [66]. Pellicle formation has been reported to modify surface characteristics and may partially mask the influence of substrate roughness on bacterial colonization [66,67]. Pellicle formation may smooth and homogenize the surface, masking the effects of Ra [46]. Additionally, material composition may influence bacterial metabolic activity and extracellular matrix production. Reduced metabolic activity within biofilms, referred to as metabolic “dormancy,” may also contribute to these observations [68]. Another proposed mechanism suggests that increased protein adsorption on rough surfaces can create an intermediate layer between bacteria and the substrate, potentially reducing bacterial attachment to nanostructured surfaces [69,70]. Therefore, the weak association observed between Ra and OD values in the present study may reflect the combined effects of surface chemistry, pellicle formation, and microbial interactions rather than the isolated influence of surface roughness.

The present study systematically examined the combined influence of build orientation, layer thickness, and polishing protocols on surface roughness and *S. mutans* adhesion on splints. The results indicate that both surface roughness and bacterial adhesion are determined by multifactorial and interdependent manufacturing conditions, rather than by any single variable in isolation. Polishing does not require an additional layer and can be reapplied when necessary, making it a practical and repeatable approach in clinical settings. In the present study, polishing provided comparable surface roughness, with the C + M method producing the lowest Ra values (0.646 ± 0.059) and a clinically feasible approach for optimizing the surface properties of 3D-printed occlusal splints. This finding suggests that polishing may be a reliable surface finishing approach for routine clinical applications. The findings highlight the need to optimize multiple production parameters simultaneously in order to effectively reduce bacterial adhesion on 3D-printed occlusal splints.

This study has certain limitations that should be acknowledged. Only one 3D-printing system and a single occlusal splint resin were investigated. Furthermore, the surface treatment methods examined were limited to polishing and selected coating procedures. Future research should evaluate a broader range of printable materials, post-curing conditions, and surface finishing approaches to provide a more comprehensive understanding of their influence on the surface characteristics of 3D-printed occlusal splint materials.

## 5. Conclusions

1. Overall, these findings indicate that surface roughness is strongly influenced by polishing protocols and their interactions with layer thickness and orientation.
2. The C + M method produced the lowest Ra values and is a clinically feasible approach for optimizing the surface properties of 3D-printed occlusal splints.
3. Optical density is comparatively more stable, being affected mainly through interaction effects rather than main factors.
4. The weak correlation between Ra and OD suggests that surface roughness alone is not a strong predictor of bacterial adhesion in this material system.

## Figures and Tables

**Table 1:** Groups of specimens.

Group	Polishing	Layer Thickness	Orientation	
1	A + M	50 µm	Horizontal	50H-A
2	A + M	100 µm	Horizontal	100H-A
3	A + RC	50 µm	Horizontal	50H-B
4	A + RC	100 µm	Horizontal	100H-B
5	A + NP	50 µm	Horizontal	50H-C
6	A + NP	100 µm	Horizontal	100H-C
7	C + M	50 µm	Horizontal	50H-D
8	C + M	100 µm	Horizontal	100H-D
9	A + M	50 µm	Vertical	50V-A
10	A + M	100 µm	Vertical	100V-A
11	A + RC	50 µm	Vertical	50V-B
12	A + RC	100 µm	Vertical	100V-B
13	A + NP	50 µm	Vertical	50V-C
14	A + NP	100 µm	Vertical	100V-C
15	C + M	50 µm	Vertical	50V-D
16	C + M	100 µm	Vertical	100V-D

**Table 2:** Effect of polishing, layer thickness, and orientation on Ra values.

Ra									
Layer Thickness	Orientation	Polishing				Total		Q	*p*
		A + M	A + RC	A + NP	C + M				
50	Horizontal	0.687 ± 0.107	0.765 ± 0.196	1.999 ± 0.304	0.646 ± 0.059	0.965 ± 0.129	Polishing	15.3	**<0.001**
	Vertical	0.546 ± 0.048	0.836 ± 0.115	2.095 ± 0.171	0.679 ± 0.112	0.982 ± 0.114	Layer Thickness	1.22	0.272
	Total	0.603 ± 0.059 ^D^	0.765 ± 0.110 ^ACD^	2.092 ± 0.168 ^B^	0.640 ± 0.051 ^AD^	0.973 ± 0.086	Orientation	3.52	0.063
100	Horizontal	0.481 ± 0.075	0.511 ± 0.037	2.071 ± 0.238	0.451 ± 0.023	0.827 ± 0.132	Polishing and Layer Thickness	19.7	**<0.001**
	Vertical	0.640 ± 0.114	1.473 ± 0.456	5.714 ± 0.759	0.504 ± 0.113	1.830 ± 0.428	Polishing and Orientation	14	**<0.001**
	Total	0.544 ± 0.056 ^ACD^	0.876 ± 0.234 ^D^	3.843 ± 0.582 ^B^	0.439 ± 0.022 ^CD^	1.209 ± 0.195	Layer Thickness and Orientation	1.69	0.176
Total	Horizontal	0.581 ± 0.063 ^E^	0.587 ± 0.101 ^E^	2.092 ± 0.181 ^F^	0.534 ± 0.034 ^E^	0.896 ± 0.092	Polishing and Layer Thickness and Orientation	16.1	**<0.001**
	Vertical	0.567 ± 0.051 ^E^	1.057 ± 0.223 ^E^	3.800 ± 0.592 ^F^	0.566 ± 0.079 ^E^	1.293 ± 0.190			
	Total	0.574 ± 0.041 ^x^	0.786 ± 0.124 ^x^	2.815 ± 0.341 ^y^	0.542 ± 0.035 ^x^	1.020 ± 0.094			

*p*—one-way analysis of variance (ANOVA). Different superscript letters indicate significant difference according to multiple comparison test (*p* < 0.001).

**Table 3:** The mean optical density (OD_630_) readings and the standard deviations determined for *S. mutans* adhesion across subgroups of each group.

OD									
Layer Thickness	Orientation	Polishing				Total		Q	*p*
		A + M	A + RC	A + NP	C + M				
50	Horizontal	0.258 ± 0.003 ^GK^	0.447 ± 0.052 ^GJ^	0.267 ± 0.024 ^IJK^	0.410 ± 0.041 ^GJ^	0.332 ± 0.020	Polishing	2.36	0.078
	Vertical	0.401 ± 0.063 ^G^	0.352 ± 0.040 ^GJ^	0.345 ± 0.045 ^IJ^	0.348 ± 0.035 ^GJK^	0.348 ± 0.021	Layer Thickness	0.466	0.496
	Total	0.312 ± 0.035	0.389 ± 0.030	0.292 ± 0.022	0.371 ± 0.024	0.341 ± 0.015	Orientation	0.266	0.607
100	Horizontal	0.291 ± 0.018 ^G^	0.350 ± 0.038 ^GH^	0.351 ± 0.045 ^IJ^	0.454 ± 0.048 ^GJ^	0.352 ± 0.021	Polishing and Layer Thickness	1.8	0.105
	Vertical	0.388 ± 0.057 ^G^	0.340 ± 0.040 ^GIJ^	0.375 ± 0.041 ^HK^	0.378 ± 0.062 ^G^	0.359 ± 0.024	Polishing and Orientation	8.84	**<0.001**
	Total	0.325 ± 0.026	0.341 ± 0.026	0.356 ± 0.028	0.409 ± 0.039	0.355 ± 0.015	Layer Thickness and Orientation	0.292	0.831
Total	Horizontal	0.268 ± 0.005 ^M^	0.388 ± 0.029 ^N^	0.296 ± 0.022 ^MN^	0.429 ± 0.031 ^LN^	0.343 ± 0.015	Polishing and Layer Thickness and Orientation	6.55	**<0.001**
	Vertical	0.385 ± 0.041 ^LMN^	0.342 ± 0.027 ^LMN^	0.351 ± 0.028 ^LMN^	0.350 ± 0.034 ^LMN^	0.354 ± 0.016			
	Total	0.318 ± 0.023	0.365 ± 0.020	0.324 ± 0.019	0.389 ± 0.023	0.348 ± 0.011			

*p*—one-way analysis of variance (ANOVA). Different superscript letters indicate significant difference according to multiple comparison test (*p* < 0.001).

**Table 4:** Correlation between surface roughness (Ra) and *S. mutans* adhesion (OD).

Independent Variable	OD	
	r	*p*
Ra	−0.155	0.050

r: Spearman correlation.

## Data Availability

The original contributions presented in this study are included in the article. Further inquiries can be directed to the corresponding author.
